# *Leishmania tropica* and *Leishmania infantum* infection in dogs and cats in central Israel

**DOI:** 10.1186/s13071-022-05272-0

**Published:** 2022-05-10

**Authors:** Gad Baneth, Yaarit Nachum-Biala, Offir Adamsky, Idit Gunther

**Affiliations:** The Koret School of Veterinary Medicine, The Hebrew University of Jerusalem, P. O. Box 12, 761001 Rehovot, Israel

**Keywords:** Cat, Dog, Israel, *Leishmania tropica*, *Leishmania infantum*, Middle East

## Abstract

**Background:**

Three species of *Leishmania* cause disease in humans in Israel and are endemic in the Middle East: *Leishmania infantum*, *Leishmania tropica* and *Leishmania major*. These species infect dogs and cats, but little is known about their prevalence in pet populations and their clinical manifestations. A study on dog and cat *Leishmania* infection was conducted in a focus of human *L. tropica* infection in central Israel with the aim of getting insight on leishmaniosis in pets in an area where human infection is highly prevalent.

**Methods:**

Blood, demographic and clinical data were collected from dogs and cats brought for veterinary care in a focus of human *L. tropica* infection during 2018–2020. kDNA PCR and internal transcribed spacer1 high-resolution melt analysis PCR (ITS1 HRM PCR) with DNA sequencing were performed for the detection of *Leishmania* and species determination.

**Results:**

Forty-three of 189 dogs (22.8%) and 44 of 152 cats (28.9%) were positive for *Leishmania* spp. infection by kDNA PCR. The ITS1 HRM PCR detected six dogs (3.3%) infected with *L. infantum* and one (0.5%) with *L. tropica,* whereas six cats (3.9%) were found infected by *L. infantum* and five (3.3%) by *L. tropica*. Four of the five *L. tropica*-positive cats suffered from weight loss, four had azotemia, two with mild and two with severe azotemia and progressive renal disease. Three cats had gingivostomatitis; three had skin lesions with abscess and ulcers in two and scales and hair loss in another cat, which was also FIV +. This is the first report of feline *L. tropica* infection in Israel. Clinical information on cats with this infection from previous studies elsewhere is scarce.

**Conclusions:**

A high rate of *Leishmania* spp. infection, mostly estimated as sub-clinical, was found in dogs and cats admitted for veterinary care in an *L. tropica* focus. Among the animals in which infection could be characterized to the species level, more dogs were infected with *L. infantum* than with *L. tropica* while 5 of 11 cats were infected with *L. tropica* and had signs of systemic and skin disease not described before in feline *L. tropica* infection.

**Graphical Abstract:**

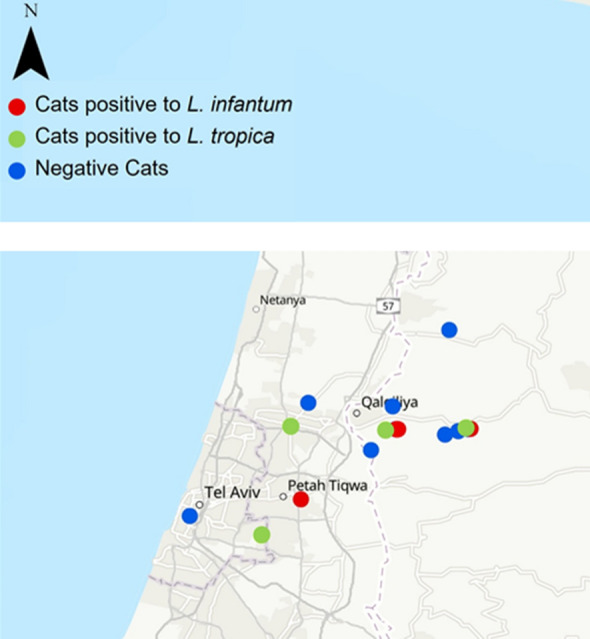

**Supplementary Information:**

The online version contains supplementary material available at 10.1186/s13071-022-05272-0.

## Background

*Leishmania* infection in Israel and the Middle East is caused in humans mainly by three different species, *Leishmania major* and *L. tropica*, which cause cutaneous leishmaniosis, and *L. infantum*, which is the main causative agent of visceral leishmaniosis. In Israel, *L. major* infection in people is present mostly in the southern arid part of the country, while *L. tropica* and *L. infantum* are prevalent in the central and northern parts where there are Mediterranean and semi-arid climates [1]. Each of these species has animal reservoirs in Israel, with rodents including the fat sand rat *Psamommys obesus* as main reservoirs for *L. major*, the rock hyrax *Procavia capensis* as the main reservoir for *L. tropica* and canines including the domestic dogs and wild canids as reservoirs for *L. infantum* [1].

At least 13 species of *Leishmania* have been reported to infect dogs worldwide including the recent detection of *L. tarentolae* infection in dogs in Italy [2, 3]. In the Middle East, dogs suffer from clinical leishmaniosis mostly caused by *L. infantum* and more rarely due to *L. major* and *L. tropica* [4–6].

Cats have been reported to be infected with seven species of the genus *Leishmania* in different parts of the world including *L. infantum*, *L. major*, *L. tropica*, *L. mexicana*, *L. venezuelensis*, *L. braziliensis* and *L. amazonensis* [7, 8]. When focusing on the Middle East and on studies in which the infecting *Leishmania* spp. were investigated, *L. infantum*, *L. major* and *L. tropica* have been reported in surveys of cat populations in Turkey and *L. infantum* and *L. tropica* in Iran [7, 9–12].

Although a few case descriptions of dogs with cutaneous leishmaniosis due to *L. tropica* and *L. major* have been published [5, 13, 14], and cat infection with *L. infantum* has also been described in a kennel in Israel where dogs and cats were housed together [15], there is no information about the prevalence of different species of *Leishmania* in dog and cat populations in Israel. A study on dog and cat infection with *Leishmania* spp. was therefore conducted in a focus of human *L. tropica* infection in central Israel with the aim of obtaining better insight into possible *L. tropica* infection in pets in an area where human infection is highly prevalent.

## Methods

### Sampled animals

Dogs and cats brought for medical treatment to a veterinary clinic in the town of Alfei Menashe in the Shomron region in Israel during 2018–2020 were included in the study. Details collected on the animals included age, breed, sex, neutering status, location of habitation, reason for arriving to the clinic, physical examination findings and results of blood tests performed at the clinic. Blood was taken from the animals by venepuncture of the cephalic or jugular veins into EDTA tubes for PCR. This study was approved by the Internal Research Committee of the Koret School of Veterinary Medicine Veterinary Teaching Hospital 2018 (KSVM-VTH/7_2018) and included residual samples from blood collected for routine testing as a part of the animal’s diagnostic procedures.

### DNA extraction, PCR for *Leishmania* and DNA sequencing

DNA was extracted from 200 µl of EDTA-anticoagulated blood samples from the dogs using the Illustra blood genomic Prep Mini Spin Kit (GE Health care, Buckinghamshire, UK), following the manufacturer’s instructions. *Leishmania* detection was performed by real-time PCR using primers JW11/JW12 targeting a 120-bp sequence of the *Leishmania* short fragment from the kinetoplast minicircle (kDNA) [16]. The kDNA PCR was performed in a total volume of 20 μl containing 3 μl DNA, 400 nM of each primer, 10 μl Maxima Hot Start PCR Master Mix (2×) (Thermo Scientific, Epsom, Surrey, UK), 50 μM of SYTO9 solution (Invitrogen, Carlsbad, CA, USA) and sterile DNase/RNase-free water (Sigma, St. Louis, MO, USA), using the StepOnePlus real-time PCR thermal cycler (Applied Biosystems, Foster City, CA, USA). Initial denaturation for 5 min at 95 °C was followed by 40 cycles of denaturation at 95 °C for 5 s, annealing and extension at 60 °C for 20 s, and final extension at 72 °C for 10 s. Amplicons were subsequently subjected to a melt step with the temperature raised to 95 °C for 10 s and then lowered to 60 °C for 1 min. The temperature was then raised to 95 °C at a rate of 0.3 °C per second. In addition, detection and identification of all samples were also carried out by PCR using primers ITS-219F and ITS-219R to amplify a 265-bp fragment of the *Leishmania* ribosomal operon internal transcribed spacer 1 (ITS1) region and then evaluated by ITS1 high-resolution melt (HRM) analysis (*Leishmnia* ITS1 HRM PCR) [17]. PCR was performed using the StepOnePlus real-time PCR thermal cycler (Applied Biosystems, Foster City, CA, USA) as previously described [18]. The *Leishmnia* ITS1 HRM PCR was performed in a total volume of 20 μl containing 4 μl DNA, 200 nM of each primer, 10 μl Maxima Hot Start PCR Master Mix (2×) (Thermo Scientific, Epsom, Surrey, UK), 50 μM of SYTO9 solution (Invitrogen, Carlsbad, CA, USA) and sterile DNase/RNase-free water (Sigma, St. Louis, MO, USA), using the StepOnePlus real-time PCR thermal cycler (Applied Biosystems, Foster City, CA, USA). Initial denaturation for 5 min at 95 °C was followed by 45 cycles of denaturation at 95 °C for 5 s, annealing and extension at 60 °C for 20 s and final extension at 72 °C for 10 s. Amplicons were subsequently subjected to a HRM step with the temperature raised to 95 °C for 10 s and then lowered to 60 °C for 1 min. The temperature was then raised to 95 °C at a rate of 0.3 °C per second. Amplification and HRM profiles were analysed using the StepOnePlus series. DNA extracted from parasite promastigote culture of *L. infantum* was used as positive control for PCR and DNA from colony-bred dogs negative by PCR for vector-borne pathogens including *L. infantum* was used as a negative control. A non-template control (NTC) with the same reagents described above but without DNA was added to each PCR to rule out contamination. All samples were tested in duplicates.

Positive *Leishmnia* ITS1 HRM PCR and kDNA amplicons were purified (EXO-Sap, New England Biolabs Inc., Ipswich, MA, USA) and DNA was sequenced at the Center for Genomic Analyses at the Hebrew University (Jerusalem, Israel) using the Big Dye Terminator cycle from Applied Biosystems ABI3700 DNA Analyzer. The ABI Data Collection and Sequence Analysis software (ABI, Carlsbad, CA, USA) was used for analysis. DNA sequences were compared to other sequences deposited in GenBank using the BLASTn website hosted by NCBI, National Institutes of Health, USA (http://www.ncbi.nlm.nih.gov). A sample was considered positive if it was positive by PCR followed by DNA sequencing and BLASTn identifying a *Leishmania* sp. sequence.

### Phylogenetic analysis

Phylograms of the 217-bp leishmanial ITS1 fragments from the dogs and cats in this study were constructed to compare these sequences to other *Leishmania* spp. present in GenBank.

Following nucleotide sequence alignment using MUSCLE, maximum likelihood (ML) tree was inferred using MEGA version 11 [19]. After creation of an alignment of 217 bp in length, a ML tree was constructed based on the Kimura two-parameter model [20] as estimated by using Aikaikeʼs information criterion (AIC). The complete deletion option resulted in 101 positions in the final dataset. Percentages of replicate trees were determined by 1000 bootstrap replicates (bs) and shown next to branches. A bs ≥ 70 was considered to provide strong support. The tree was composed of 38 nucleotide sequences of analysed and relevant *Leishmania* species and including a *Trypanosoma cruzi* ITS1 sequence as outgroup.

### Statistical analysis

The chi-square test was used for ordinal variables. The continuity correction was used when 2 × 2 tables were used. Statistical analysis was performed using the statistics software SPSS(R) 26.0 software (IBM, Armonk, New York, USA). Statistical significance was defined as *P* < 0.05. Maps showing the collection sites were constructed using the ArcGIS Pro 2.8.4 software (Esri, Redlands, CA, USA).

## Results

Samples from 189 dogs and 152 cats brought for medical treatment to a veterinary clinic in the town of Alfei Menashe during 2018–2020 were included in the study. The prevalence of infection by the kDNA PCR for *Leishmania* spp. was 43/189 (22.8%) for dogs and 44/152 (28.9%) for cats (Table 1). The ITS1 HRM PCR, which is less sensitive than the kDNA, but is able to distinguish between *Leishmania* spp., showed that seven dogs (3.7%) were positive of which six (3.3%) were infected with *L. infantum* and one (0.5%) with *L. tropica* (Additional file 1: Table S1). Eleven cats (7.2%) were positive by the ITS1 HRM PCR, of which six (3.9%) were infected by *L. infantum* and five (3.3%) by *L. tropica*. In total, there were 18 (5.3%) animals (dogs and cats) of 341 that were positive by the ITS1 HRM PCR for *Leishmania* spp., of which 12 (3.5%) were positive for *L. infantum* and 6 (1.85%) were positive for *L. tropica*. All the six *L. tropica* sequences were identical to each other and 100% identical to GenBank accession MN891726.1 from a human in Pakistan. Eleven of the *L. infantum* sequences from dogs and cats were 98.5–100% identical to each other, and only one (cat 319) was 92.7–93.2% identical to the other *L. infantum* sequences. The 11 *L. infantum* sequences were 98.5–100% identical to GenBank accession MT416148.1 of *L. infantum* from Italy, which was the first hit by BLASTn search. New ITS1 sequences from this study were deposited in GenBank (accession numbers ON036070-ON036087) (Additional file 1: Table S1).

**Table 1:** Prevalence of infection with *Leishmania* spp. in dogs and cats

	ITS1 HRM PCR+ (%)	*Leishmania infantum* according to ITS1 HRM PCR and DNA sequencing (%)	*Leishmania tropica *according to ITS1 HRM PCR and DNA sequencing (%)	kDNA+ (%)
Dogs	7/189 (3.7)	6/189 (3.2%)	1/189 (0.5)	43/189 (22.7)
Cats	11/152 (7.2)	6/152 (3.9)	5/152 (3.3)	44/152 (28.9)
	χ^2^ = 1.456, *df* = 1, *P* = 0.228	χ^2^ = 0.008, *df* = 1, *P* = 0.929	χ^2^ = 3.902, *df* = 2, *P* = 0.142	χ^2^ = 1.391, *df* = 1, *P* = 0.238

There was no significant difference between the prevalence of *Leishmania* spp. infection in the dog and cat groups by kDNA PCR (*χ*^2^ = 1.391, *df* = 1, *P* = 0.238) and also by the ITS1 HRM PCR (*χ*^2^ = 1.456, *df* = 1, *P* = 0.228). There was also no significant difference between the prevalence of *L. tropica* infection in cats (5/152) and dogs (1/189) (*χ*^2^ = 2.288, *df* = 1, *P* = 0.130) and *L. infantum* infection in cats (6/152) and dogs (6/189) (*χ*^2^ = 0.008, *df* = 1, *P* = 0.929).

**Fig. 1:**
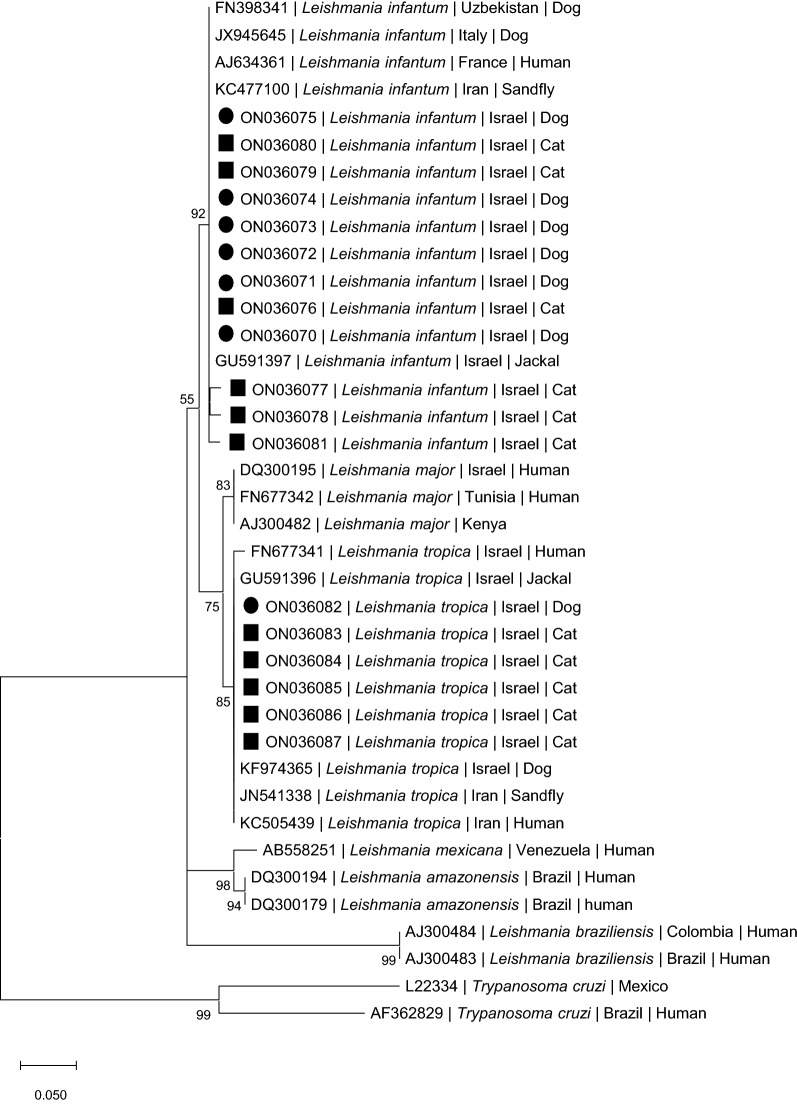
A maximum likelihood phylogram comparing 217-bp DNA sequences of the ribsomal ITS1 locus from the dogs and cats included in the study and other *Leishmania* spp. New sequences derived from this study are marked with black squares for dogs and black circles for cats. The GenBank accession numbers, species of infected host (when known) and country of origin are included for each sequence. The tree is drawn to scale, with branch lengths measured in the number of substitutions per site. Bootstrap values > 50% are indicated

Phylogenetic analysis of the positive ITS1 DNA sequences (Fig. 1) indicated that the dog and cat *L. infantum* sequences clustered with each other and also with strong support with *L. infantum* sequences deposited in GenBank from humans, dogs and sand flies from Europe and Asia, and with a sequence of *L. infantum* from an Israeli jackal. The *L. tropica* sequences from five cats and one dog from this study clustered with strong support with *L. tropica* sequences deposited in GenBank from a human and a sand fly from Iran and from a dog, human and golden jackal in Israel.

**Fig. 2:**
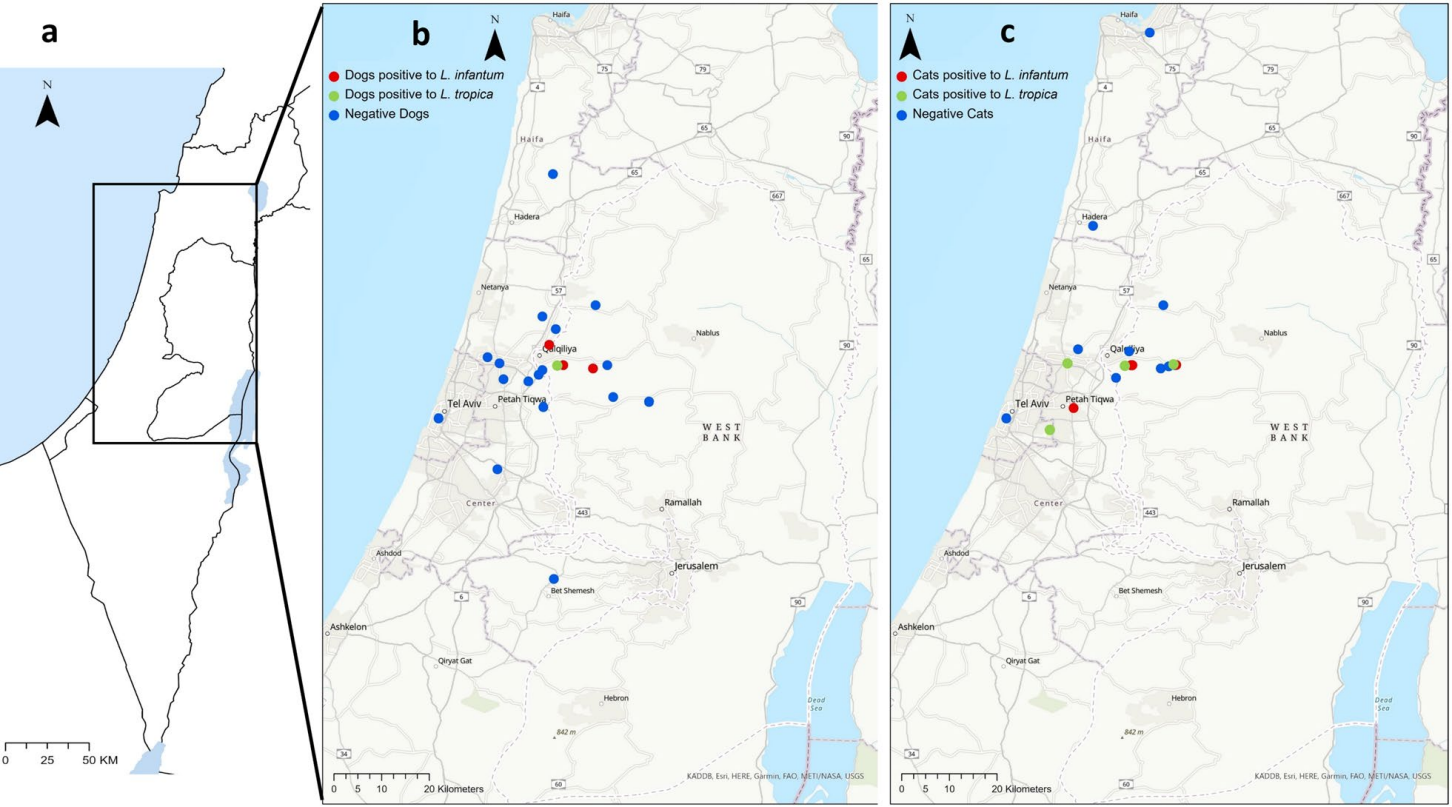
The location of the study area on a map of Israel (**a**), the locations of surveyed dogs (**b**) and cats (**c**) with locations of animals positive for *L. infantum* marked in red circles and for those positive for *L. tropica* in green

The dogs and cats that were positive for *L. infantum* or *L. tropica* by PCR were located in the same area in central Israel (Fig. 2). The animals were divided into breed categories (purebred and mixed-breed), age groups (< 12 months; > 12 months) and 2 geographic regions: the Sharon region in the coastal plain of central Israel and the more inland and hilly Shomron to the east of the Sharon. There were some missing data points for some of the variables. As there was only one dog that was positive for *L. tropica* by ITS1 HRM PCR, all the *Leishmania* spp. PCR-positive dogs were analyzed together. There was no statistically significant association between PCR positivity by kDNA PCR or by ITS HRM PCR and the dog sex, breed, neuter status, age group or geographic region of habitation (Additional file 1: Table S2). When grouping all the PCR-positive cats together, there was also no statistically significant association with sex, breed, neuter status, age group and geographic region of habitation for the cats (Additional file 1: Table S3).

Clinical evaluation of the data on the five cats infected with *L. tropica* according to the ITS1 HRM PCR showed that they ranged in age from 1 to 9.5 years, three were males and two were females, all were of mixed breed and neutered, three of them were negative for the feline leukemia virus (FeLV) and feline immunodeficiency virus (FIV) tested using the SNAP FIV/FeLV Combo Test (Idexx, Westbrook, ME, USA), one was positive for FIV, and another was not tested for retroviral infection. Four of the five cats suffered from weight loss and three had gingivostomatitis. Three cats had skin lesions, the FIV-positive cat had excessive scales and hair loss, and two other cats had skin ulcers and abscesses over their limbs. All four cats who had a partial or full serum biochemistry panel had azotemia, two with mild azotemia (creatinine > 1.6 mg/dl) and two others with severe azotemia (creatinine > 7 mg/dl).

The six cats with *L. infantum* infection ranged in age from 6 months to 16.5 years, included five females and one male; one was a Siamese while the others were of mixed breed. Three females were presented for routine sterilization, of which two had no clinical signs of disease and one was blind because of earlier bilateral corneal perforation. One cat tested FIV-positive, two were negative for FIV and FeLV, and the three others were not tested for retroviral infection. Two cats had gingivostomatitis, two had urinary tract infections, and one was severely azotemic with kidney disease. Another cat presented with acute anorexia and cough due to lower respiratory disease.

The dog infected with *L. tropica* was 5 years old and admitted for periodontal disease. It had a low body condition score and leukopenia. The six dogs infected with *L. infantum* ranged from 4 to 12 years of age, three were females and three were males, two dogs were purebred and four were of mixed breed, one had a co-infection with *Ehrlichia canis* confirmed by PCR, three had suffered from weight loss, two had skin lesions, and two had severe azotemia with kidney disease. When comparing the sex, breed, neuter status, age group and geographic region of habitation between all the dogs infected by *L. infantum* and the dog infected with *L. tropica* (Additional file 1: Table S4), and also separately between all the cats infected by these two species (Additional file 1: Table S5), there were no statistically significant associations between any of these variables and the animals infected with each of these *Leishmania* spp.

## Discussion

The results of this study show that *Leishmania* infection is widespread in both the canine and feline populations in a focus of cutaneous human leishmaniosis in central Israel. The kDNA PCR results showed that 22.7% of the dogs and 28.9% of the cats brought for veterinary care were positive for *Leishmania* spp. Although this sample of animals does not represent the total population of dogs and cats in the area, as most animals tested were brought because of suspected illness or prior to medical procedures such as neuter surgery and dental hygiene, it does provide important information about the spread of this infection. It is likely that most of the PCR-positive animals were only infected sub-clinically, as surveys from other areas of the world have shown that *Leishmania* PCR-positive dogs and cats were mostly infected asymptomatically [21–24]. However, some of the infected animals in the current study had clinical findings compatible with leishmaniosis such as kidney disease and skin lesions [8, 25]. The smaller ratio of dogs and cats that were positive by ITS1 HRM PCR amounted to 16.3% and 24.9% of the kDNA-positive animals, respectively. The lower sensitivity of the ITS1 HRM PCR is due the fact that the kDNA minicircle sequence targeted by the kDNA PCR has about 10,000 copies per individual *Leishmania* parasite while there are only 40–200 copies of the ITS1 region of the rRNA gene targeted by the ITS1 HRM PCR [26]. Despite this, the ITS1 HRM PCR provided valuable information about the identity of the *Leishmania* species which are circulating among the infected animals in the study area. The ITS1 sequences of *L. tropica* and *L. infantum* derived from this study are identical or almost identical to other sequences of these species deposited in GenBank and clustered with strong support by phylogenetic analysis with sequences from GenBank (Fig. 1).

Other studies on canine and feline leishmaniosis from the Middle East have found variable infection rates in different countries and areas using PCR. Studies on canine leishmaniosis from Turkey, Iran and Saudi Arabia have reported between 1.6% and 42% positivity for *Leishmania* spp. by PCR of dogs surveyed using blood or other tissue samples [27–29]. The detection of positive kDNA PCR in 22.7% of the dogs in the current study from central Israel is therefore compatible with the infection rates found in other Middle Eastern countries.

When comparing feline leishmaniosis prevalence surveys from the Middle East, studies of cats from Turkey have shown that 0.5% of 1101 stray cats from Izmir were positive for *Leishmania* by PCR of which two cats were infected with *L. tropica*, one with *L. infantum* and three others with an undefined species [9]. Another study of cats admitted to veterinary clinics in the Ege region of Turkey found that 8.8% of 147 cats were positive for *Leishmania* by PCR, four of them (2.7%) with *L. major* and nine (6.1%) with *L. tropica* [7]. A third study in cats from animal shelters from the western Aegean region of Turkey found that 2.3% of the 386 cats were positive for *Leishmania* with one cat infected by *L. infantum* and the other cats having DNA sequences that matched *L. tropica*, *L. infantum* and *L. major* with insufficient identity to make a credible speciation [12]. In a study from the Kerman region in southeastern Iran which surveyed 180 stray cats by PCR, 25 cats (13.8%) were positive for *L. infantum* and 3 (1.7%) for *L. tropica* [10]. These studies present dissimilar results regarding the identity and proportion of infecting *Leishmania* spp. in the different areas of the Middle East probably due to different local ecological conditions and vector sand fly fauna and densities. It is interesting to note that while cats in Turkey were detected with *L. major* infection, this was not the case in Iran and in the present study from Israel. This might be due to the fact that the area sampled in central Israel is not considered endemic for *L. major* in humans [1].

Infection of dogs with *L. infantum* is well known in central Israel [1], and there have been a few reports of canine *L. tropica* infection in other parts of the country [5, 13]. However, feline *L. tropica* infection has not been described before in Israel and has rarely been reported in detail from other countries [7, 9, 10]. Although the sampling was done in a focus of human *L. tropica* infection, it is also an area which is endemic for *L. infantum* infection in dogs, and therefore the finding that the majority of ITS1 HRM PCR-positive dogs were infected with *L. infantum* is sensible. There was also one dog identified with *L. tropica* infection which had a poor body condition and periodontal disease. It was interesting to find out that almost half of the ITS1 HRM PCR-positive cats, 5 of 11, were infected with *L. tropica*. The detection of *L. tropica* infection in several cats in the study region indicates that cats are frequently exposed to this infection, can serve as sentinels for the presence of *L. tropica* and may also serve as reservoirs able to infect sand flies, as has been shown experimentally for *L. infantum* in cats in Europe and South America [30–32].

A study on risk factors for the expansion of cutaneous leishmaniosis caused by *L. tropica* in a disease focus in Morocco found that *Phlebotomus sergenti*, the main sand fly vector of *L. tropica* in Morocco and in Israel, was the most abundant sand fly species [33, 34]. Three percent of the dogs tested were positive for *L. tropica*, and cat blood meal was found in blood engorged *P. sergenti* females, as well as blood meals from humans, rabbit and birds [35]. In a study on the sand fly species composition and their host preference from the Bethlehem district of Palestine, bordering central Israel, *P. sergenti* females were found to have dog and cat blood meals, as well as blood meals of other hosts [36]. Although no cats were surveyed in these studies from Morocco and Palestine, they confirmed that *P. sergenti* females feed on cats and are therefore possibly capable of transmitting *L. tropica* to and from cats.

The clinical manifestations of *L. tropica* infection in cats are not known. There are detailed descriptions of *L. tropica* infection in humans where it causes mostly cutaneous disease and has also been incriminated as a cause of visceral leishmaniosis [37–41]. A similar situation exists in dogs where *L. tropica* causes cutaneous disease and has also been described to cause visceral disease in some dogs with lesions which are similar to those found in canine *L. infantum* infection [4, 6, 42–44]. Three studies that reported *L. tropica* infection in cats from the Aegean region in Turkey did not provide data on the clinical signs found in these cats [7, 9, 12]. In the study from the Kerman region in Iran [10], the three *L. tropica*-positive cats found had a clinical status that was defined as clinically suspected and one was FIV + but no other specific clinical findings were described. To our knowledge, the clinical findings reported in five cats infected with *L. tropica* in the current study are therefore the first more detailed description of disease in cats with this infection. Although there were a variety of clinical signs in these cats, and other conditions are possibly responsible for some of these clinical signs, four of the five cats suffered from weight loss, four had azotemia, of which two had severe azotemia and progressive renal disease, and three had skin lesions, consisting of limb wound dermal ulceration and abscess or hair loss and scales. While it is difficult to draw conclusions about the clinical signs associated with *L. tropica* from only five cats, it is clear that they were debilitated and suffered from a generalized disease with at least two cats having severe kidney dysfunction. Renal disease is frequent in dogs with *L. infantum* infection because of immune-complex glomerulonephritis and has also been reported in feline leishmaniosis due to *L. infantum* [8, 24]. If *L. tropica* is able to visceralize in cats as it does in humans and dogs, it is likely that the kidneys would be affected. Nonetheless, more clinical and pathological studies are needed to confirm this.

This study had several limitations, which included a relatively small number of dogs and cats that were surveyed and missing data points on some of the animals. As the study was based on animals that were admitted to a private veterinary clinic, testing for some concomitant disease conditions and exhaustive medical investigation were in some cases not possible, because they depended on the owner’s decision and willingness to pursue further testing. Despite this, the study confirms infection with *L. tropica* and *L. infantum* in dogs and cats in central Israel and adds to our knowledge about their clinical presentation. Further studies are needed to understand the pathogenesis and clinical implications of feline and canine *L. tropica* infection and the possible role of cats and dogs as reservoirs and sentinels for infection.

## Conclusions

A high rate of *Leishmania* spp. infection was found in dogs and cats admitted for veterinary care in a focus of human *L. tropica* infection in Israel. PCR allowing *Leishmania* species determination indicated that *L. infantum* was prevalent in the majority of dogs in which infection could be characterized to the species level, and almost a half of the cats with characterized infection were infected with *L. tropica* and had signs of systemic and skin disease, which to our knowledge have not described before in feline *L. tropica* infection.

## Supplementary Information

**Additional file 1: Table S1.** New GenBank accessions generated in the study with details on animal host, closest GenBank accession and species identification.

## Data Availability

All data generated or analysed during this study are included in this published article.
