# Supplementary material for: Leishmania tropica and Leishmania infantum infection in dogs and cats in central Israel
Source: Parasit Vectors. 2022 May 10;15:147. doi: 10.1186/s13071-022-05272-0 (PMC9087926; doi:10.1186/s13071-022-05272-0)
Supplement: Supplementary file 1 — Additional file 1: Table S1. New GenBank accessions generated in the study with details on animal host, closest GenBank accession and species identification. [file 13071_2022_5272_MOESM1_ESM.docx]

Additional file1: Table S1 – New GenBank accessions generated in the study with details on animal host, closest GenBank accession and species identification.

| **New GenBank accession number** | **Sample designation and animal host** | **Closest GenBank accession**  (accession number, % identity, % cover) | ***Leishmania* species identification** |
| --- | --- | --- | --- |
| ON036070 | HU:Dog8 | MT416148.1 (100%, 100%) | *L. infantum* |
| ON036071 | HU:Dog17 | MT416148.1 (100%, 100%) | *L. infantum* |
| ON036072 | HU:Dog48 | MT416148.1 (100%, 100%) | *L. infantum* |
| ON036073 | HU:Dog195 | MT416148.1 (100%, 100%) | *L. infantum* |
| ON036074 | HU:Dog208 | MT416148.1 (100%, 100%) | *L. infantum* |
| ON036075 | HU:Dog218 | MT416148.1 (98.5%, 100%) | *L. infantum* |
| ON036076 | HU:Cat7 | MT416148.1 (99.5%, 100%) | *L. infantum* |
| ON036077 | HU:Cat311 | MH231229.1 (99.5%, 100%) | *L. infantum* |
| ON036078 | HU:Cat 321 | MT416148.1 (99.5%, 100%) | *L. infantum* |
| ON036079 | HU:Cat322 | MT416148.1 (100%, 100%) | *L. infantum* |
| ON036080 | HU:Cat332 | MT416148.1 (99.5%, 100%) | *L. infantum* |
| ON036081 | HU:Cat319 | MT416148.1 (93.2%, 100%) | *L. infantum* |
| ON036082 | HU:Dog57 | MN891726.1 (100%, 100%) | *L. tropica* |
| ON036083 | HU:Cat359 | MN891726.1 (100%, 100%) | *L. tropica* |
| ON036084 | HU:Cat361 | MN891726.1 (100%, 100%) | *L. tropica* |
| ON036085 | HU:Cat366 | MN891726.1 (100%, 100%) | *L. tropica* |
| ON036086 | HU:Cat365 | MN891726.1 (100%, 100%) | *L. tropica* |
| ON036087 | HU:Cat367 | MN891726.1 (100%, 100%) | *L. tropica* |

Additional file1: Table S2 - Demographic characteristics of dog infection with *Leishmania* spp*.*

| **Variable** | **Number of dogs** | **PCR + (%) for *Leishmania* spp.** | |
| --- | --- | --- | --- |
|  |  | ITS+ | kDNA+ |
| **Total** | 189 | 7 | 43 |
| **Sex**  Female  Male | 98  91 | 3 (3.1)  4 (4.4)  χ2=0.100, *df*=1, *P*=0.920 | 20 (20.4)  23 (25.3)  χ2=0.005, *df*=1, *P*=0.944 |
|  |  |  |  |
|  |  |  |  |
| **Breed**  Purebred  Mixed | 103  86 | 2 (1.9)  5 (5.8)  χ2=1.034, *df*=1, *P*=0.309 | 21 (20.4)  22 (25.6)  χ2=0.454, *df*=1, *P*=0.500 |
|  |  |  |  |
|  |  |  |  |
| **Sterilization**  Yes  No | 132  50 | 6 (4.5)  1 (2.0)  χ2=0.114 *df*=1, *P*=0.736 | 31 (23.5)  12 (24.0)  χ2=0.0001, *df*=1, *P*=1 |
| **Age**  <12 months  >12 months | 40  140 | 0 (0)  7 (5)  χ2=0.850, *df*=1, *P*=0.349 | 11 (27.5)  32 (22.9)  χ2=0.305, *df*=1, *P*=0.581 |
| **Geographic area**  Shomron  Sharon  Other | 160  26  3 | 7 (4.4)  0 (0)  0 (0)  χ2=1.318, *df*=2, *P*=0.517 | 35 (21.9)  7 (26.9)  1 (33.3)  χ2=0.519, *df*=2, *P*=0.772 |

Additional file1: Table S3 - Demographic characteristics of cat infection with *Leishmania* spp*.*

| **Variable** | **Number of cats** | **PCR + (%)** | |
| --- | --- | --- | --- |
|  |  | ITS+ | kDNA+ |
| **Total** | 152 | 11 | 44 |
| **Sex**  Female  Male | 66  86 | 7 (10.6)  4 (4.7)  χ2=1.185, *df*=1, *P*=0.276 | 20 (30.3)  24 (27.9)  χ2=0.020, *df*=1, *P*=0.887 |
| **Breed**  Purebred  Mixed | 17  135 | 1 (5.9)  10 (7.4)  χ2=0.0001, *df*=1, *P*=1 | 4 (23.5)  40 (29.6)  χ2=0.057, *df*=1, *P*=0.811 |
| **Sterilization**  Yes  No | 110  42 | 10 (9.1)  1 (2.4)  χ2=1.162, *df*=1, P=0.281 | 32 (29.1)  12 (28.6)  χ2=0.0001, *df*=1, P=1 |
| **Age**  <12 months  >12 months | 24  112 | 1 (4.2)  9 (8.0)  χ2=0.052, *df*=1, *P*=0.820 | 9 (37.5)  30 (26.8)  χ2=0.647, *df*=1, *P*=0.421 |
| **Geographic area**  Shomron  Sharon | 137  15 | 8 (5.8)  3 (20.0)  χ2=2.204, *df*=2, *P*=0.138 | 42 (30.7)  2 (13.3)  χ2=1.220, *df*=2, *P*=0.269 |

Additional file1: Table S4 – Comparison of demographic characteristics of dogs infected with *L. infantum* and *L. tropica.*

| **Variable** | **Number of dogs** | **ITS1 HRM PCR -positive** | |  |
| --- | --- | --- | --- | --- |
|  |  | *L. infantum* | *L. tropica* | *P* value |
| **Total** | 189 | 6 | 1 | χ2=2.329, *df*=1, *P*=0.127 |
| **Sex**  Female  Male | 98  91 | 3  3 | 0  1 | χ2=1.479, *df*=2, *P*=0.579 |
| **Breed**  Purebred  Mixed | 103  86 | 2  4 | 0  1 | χ2=2.737, *df*=2, *P*=0.254 |
| **Sterilization**  Yes  No | 132  50 | 5  1 | 1  0 | χ2=1.009, *df*=2, *P*=0.604 |
| **Age**  <12 months  >12 months | 40  140 | 0  6 | 0  6 | χ2=3.443, *df*=2, *P*=0.179 |
| **Geographic area**  Shomron  Sharon  Other | 160  26  3 | 6  0  0 | 1  0  0 | χ2=2.380, *df*=4, *P*=0.666 |

Additional file1: Table S5 – Comparison of demographic characteristics of cats infected with *L. infantum* and *L. tropica.*

| **Variable** | **Number of cats** | **ITS1 HRM PCR -positive** | |  |
| --- | --- | --- | --- | --- |
|  |  | *L. infantum* | *L. tropica* | *P* value |
| **Total** | 152 | 6 | 5 | χ2=0.0001, *df*=1, *P*=1 |
| **Sex**  Female  Male | 66  86 | 5  1 | 2  3 | χ2=4.242, *df*=2, *P*=0.120 |
| **Breed**  Purebred  Mixed | 17  135 | 1  5 | 0  5 | χ2=1.350, *df*=2, *P*=0.509 |
| **Sterilization**  Yes  No | 110  42 | 5  1 | 5  0 | χ2=3.780, *df*=2, *P*=0.151 |
| **Age**  <12 months  >12 months | 24  112 | 1  4 | 0  5 | χ2=1.992, *df*=2, *P*=0.369 |
| **Geographic area**  Shomron  Sharon | 137  15 | 5  1 | 3  2 | χ2=3.726, *df*=2, *P*=0.155 |
